# Deep Learning-Based Joint Effusion Classification in Adult Knee Radiographs: A Multi-Center Prospective Study

**DOI:** 10.3390/diagnostics14171900

**Published:** 2024-08-29

**Authors:** Hyeyeon Won, Hye Sang Lee, Daemyung Youn, Doohyun Park, Taejoon Eo, Wooju Kim, Dosik Hwang

**Affiliations:** 1 School of Electrical and Electronic Engineering, Yonsei University, Seoul 03722, Republic of Korea; hywon4825@gamil.com (H.W.); dhpark.ee@gmail.com (D.P.); ship9136@naver.com (T.E.); 2 Probe Medical Inc., 61, Yonsei-ro 2na-gil, Seodaemun-gu, Seoul 03777, Republic of Korea; 3 Independent Researcher, Seoul 06295, Republic of Korea; hs.lisa.lee@gmail.com; 4 School of Management of Technology, Yonsei University, Seoul 03722, Republic of Korea; daemyungyoon@yonsei.ac.kr; 5 Department of Industrial Engineering, Yonsei University, Seoul 03722, Republic of Korea; 6 Artificial Intelligence and Robotics Institute, Korea Institute of Science and Technology, 5, Hwarang-ro 14-gil, Seongbuk-gu, Seoul 02792, Republic of Korea; 7 Department of Oral and Maxillofacial Radiology, Yonsei University College of Dentistry, Seoul 03722, Republic of Korea; 8 Department of Radiology, Center for Clinical Imaging Data Science (CCIDS), Yonsei University College of Medical, Seoul 03722, Republic of Korea

**Keywords:** knee joint effusion, radiographs, orthopedic diagnosis, deep learning, classification, visualization

## Abstract

Knee effusion, a common and important indicator of joint diseases such as osteoarthritis, is typically more discernible on magnetic resonance imaging (MRI) scans compared to radiographs. However, the use of radiographs for the early detection of knee effusion remains promising due to their cost-effectiveness and accessibility. This multi-center prospective study collected a total of 1413 radiographs from four hospitals between February 2022 to March 2023, of which 1281 were analyzed after exclusions. To automatically detect knee effusion on radiographs, we utilized a state-of-the-art (SOTA) deep learning-based classification model with a novel preprocessing technique to optimize images for diagnosing knee effusion. The diagnostic performance of the proposed method was significantly higher than that of the baseline model, achieving an area under the receiver operating characteristic curve (AUC) of 0.892, accuracy of 0.803, sensitivity of 0.820, and specificity of 0.785. Moreover, the proposed method significantly outperformed two non-orthopedic physicians. Coupled with an explainable artificial intelligence method for visualization, this approach not only improved diagnostic performance but also interpretability, highlighting areas of effusion. These results demonstrate that the proposed method enables the early and accurate classification of knee effusions on radiographs, thereby reducing healthcare costs and improving patient outcomes through timely interventions.

## 1. Introduction

Knee effusion is a primary symptom of knee joint diseases, particularly common among patients with degenerative arthritis such as osteoarthritis [1,2,3]. Without timely detection and appropriate treatment, effusion can lead to significant consequences, causing continuous joint deterioration and impacting patients’ quality of life [4,5,6].

According to orthopedic diagnostic guidelines, identifying effusion in X-ray images involves recognizing a well-defined, rounded, homogeneous soft tissue density in the suprapatellar recess on lateral X-rays [7,8,9]. However, effusion is often challenging to discern in X-ray images, particularly in the early stages due to subtle initial signs that can be easily overlooked. While magnetic resonance imaging (MRI) offers better clarity for diagnosing knee effusion, assessing effusion in X-ray images is crucial for optimizing time and cost efficiency [10,11]. Therefore, radiographic imaging plays a pivotal role in diagnosing knee effusion [12,13,14,15].

Recent advancements in radiology have shown significant research growth, particularly in the application of artificial intelligence (AI) and deep learning for radiological evaluations and automation [16,17,18,19,20]. Notably, these advancements in X-ray imaging have shown promising results for early disease detection [21,22]. Despite the demonstrated efficacy of deep learning across various radiological applications, to our knowledge, no AI research exists for diagnosing knee effusion in X-ray images. Current studies have mainly focused on knee joint recognition and the severity assessment of knee osteoarthritis [23,24,25]. Additionally, attempts to visualize effusion areas in joints have been limited to the elbow region [26], leaving a notable gap in similar applications for knee effusion detection.

Therefore, this study proposes an AI-based diagnostic methodology that enhances orthopedic diagnoses by classifying and visualizing knee joint effusion on X-ray imaging. Our approach involves performing image-level classification of knee effusion using novel preprocessing techniques, focusing on identifying predominant effusion sites. Additionally, we visualize the effusion areas through weakly supervised localization.

## 2. Materials and Methods

### 2.1. Patient Population

**Figure 1:**
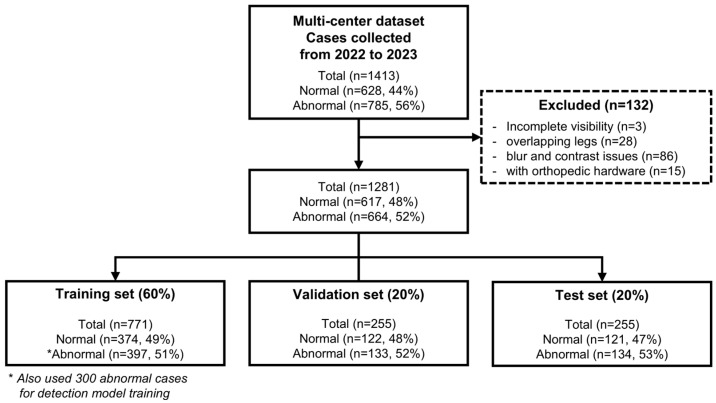
Flowchart for study inclusion and exclusion.

This multi-center prospective study was approved by the institutional review board, and written consent for all subjects was waived. We acquired X-ray images from 1413 cases from four hospitals, which were prospectively collected between February 2022 and March 2023. We excluded 132 cases based on the following criteria: (a) incomplete visibility of effusion areas, (b) overlapping left and right knees in a single radiograph, (c) images that are blurred or excessively dark or bright, and (d) presence of orthopedic hardware such as K-wires (KW) around the patella. The remaining 1281 cases were randomly divided into an 80% of training set and a 20% of test set. The data flow diagram is illustrated in Figure 1.

**Figure 2:**
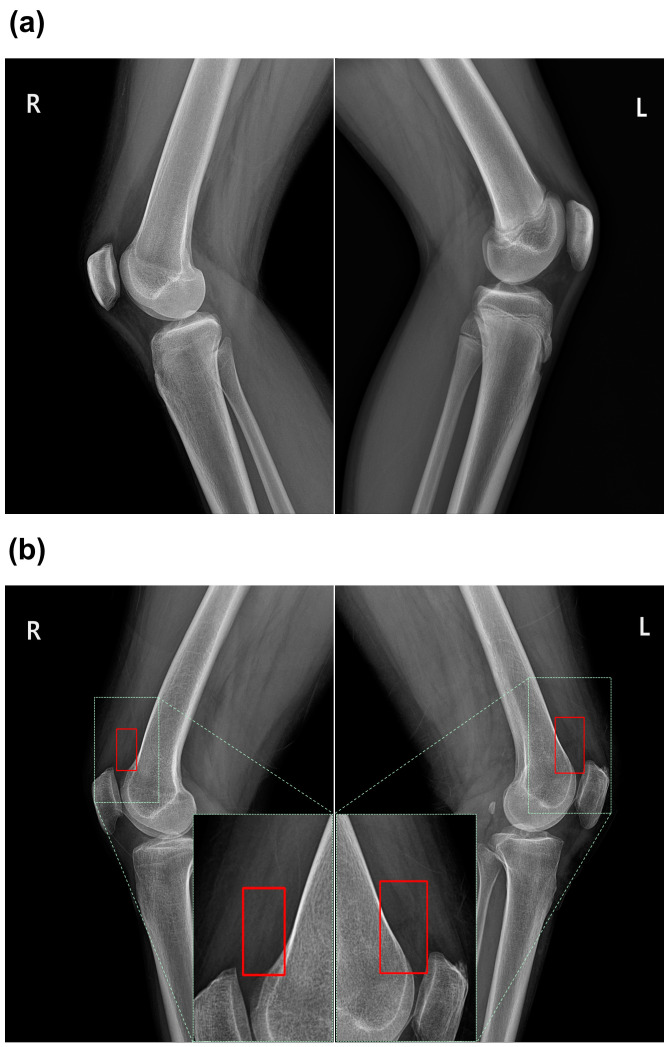
Sample X-ray images of patients with knee: (**a**) normal case; (**b**) effusion case (the red bounding box indicates the area of effusion).

As shown in Figure 1, 300 randomly selected effusion cases in the training set were annotated with bounding boxes (bbox) around the patella by a medical AI researcher to train a patella detection model. The dataset was then divided into a training set of 200 cases (67%) and a test set of 100 cases (33%). Additionally, three orthopedic physicians, each with more than 10 years of experience, annotated all cases for the presence of effusion. Effusion was defined as a well-defined, rounded, homogeneous soft tissue density within the suprapatellar recess on a lateral radiograph. Consequently, the training set included 496 (48%) normal cases and 530 (52%) effusion cases, while the test set included 121 (47%) normal cases and 134 (53%) effusion cases. Sample X-ray images of normal and effusion cases are shown in Figure 2.

### 2.2. X-ray Acquisition Parameters

The X-ray images were taken in the lateral decubitus position, and detailed information for each hospital is provided in Table 1. Due to privacy concerns, the images were collected in the Joint Photographic Experts Group (JPEG) format, limiting the availability of further details.

### 2.3. Methodology

**Figure 3:**
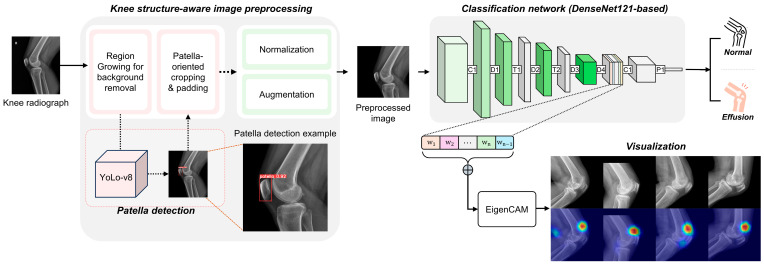
Proposed architecture for knee joint effusion classification and visualization.

We proposed a method that classifies the presence of knee effusion and enables the visualization of the effusion area. Our proposed architecture is depicted in Figure 3.

#### 2.3.1. Knee Structure-Aware Image Preprocessing

To address variations in fields of view (FoV) and intensity levels caused by different acquisition protocols across institutions, we developed a robust preprocessing strategy. First, we addressed image intensity variations by removing background elements outside the body using a region-growing algorithm. Second, we constructed a deep learning-based patella detection model using the YOLO v8 [27] architecture to crop the effusion area. To standardize each predicted bounding box (bbox) of the patella, we first aligned the center of the bbox of all data to the average center position of the patella. Then, we rescaled the image based on the smallest bbox in the training set. After scaling, we added zero-padding to ensure that the image was centered. Subsequently, the image was cropped to a size of 1600 × 1600 pixels to preserve the area information of the effusion without distortion. This process ensures a standardized input image that includes the effusion area with a uniform size. The results of our proposed preprocessing method are shown in Supplement S2.

#### 2.3.2. DL Architecture

We conducted a comparative analysis of five different network models pre-trained on ImageNet [28]: VGG19 [29], ResNet50 [30], DenseNet121 [31], EfficientNet [32], and Vision Transformer (ViT) [33]. The input consisted of preprocessed images derived from the original X-ray images, and the output was a continuous value between 0 and 1 representing the probability of effusion presence. The training set (n = 1026) was divided into a development set (n = 771, 75%) and a validation set (n = 255, 25%). For the qualitative analysis of the classification model, we compared various class activation map (CAM) methodologies and empirically selected Eigen-CAM [34] for its superior qualitative performance.

#### 2.3.3. Model Specifications

This study utilized the PyTorch 2.0 framework to train a binary classification model with CrossEntropyLoss. The model was trained for 150 epochs with a batch size of 14, a learning rate of 0.001, and the SGD optimizer. Training was performed on an NVIDIA RTX A5000 24GB GPU with CUDA version 11.8 and an AMD EPYC 7452 32-core processor, sourced from COMPUWORKS Co., Seoul, Republic of Korea, and it took approximately 40 min.

### 2.4. Statistical Analysis

For the statistical analysis, the following software was used: R Core Team, 2024 (R: A Language and Environment for Statistical Computing. R Foundation for Statistical Computing, Vienna. https://www.R-project.org, accessed on 23 April 2024). DeLong’s test [35] and McNemar’s test [36] were used to compare the performances of the two models. A *p* value of less than 0.05 was considered statistically significant.

## 3. Results

### 3.1. Performance of the Classification Models

We compared the classification performances of five different deep learning models using images without preprocessing. The DenseNet121 achieved the highest area under the receiver operating characteristic (ROC) curve (AUC) on the validation set, and the results are presented in Supplement S3. Therefore, we selected DenseNet121 as a baseline classification model to analyze the impacts of our proposed method. 

**Figure 4:**
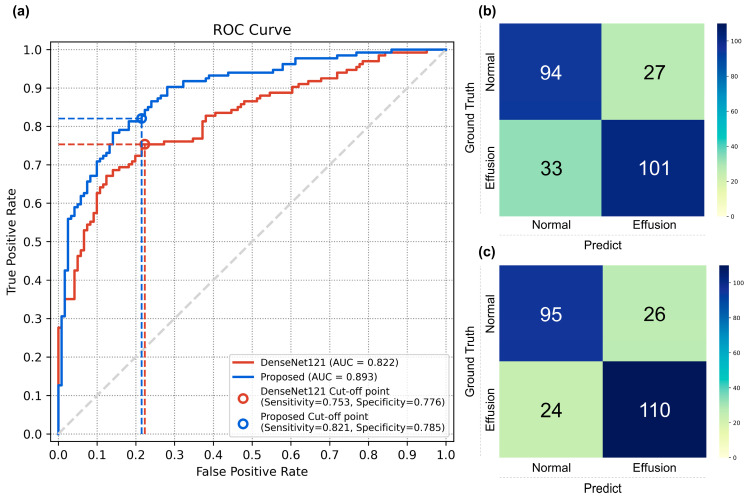
(**a**) ROC curves; (**b**) Confusion matrix of the Densenet121; (**c**) Confusion matrix of the proposed method.

In the effusion classification, the proposed method showed a significantly higher AUC (95% confidence interval [CI]) compared to the baseline DenseNet121 model, with 0.892 (0.853–0.931) versus 0.821 (0.770–0.872), with a *p*-value of <0.001. The sensitivities (95% CI) were 0.820 (0.753–0.880) and 0.753 (0.686–0.835), and the specificities (95% CI) were 0.785 (0.710–0.851) and 0.776 (0.619–0.785), respectively. The results are shown in Table 2. Figure 4 displays the ROC curves and confusion matrices.

### 3.2. Comparison with Physician Evaluation

**Figure 5:**
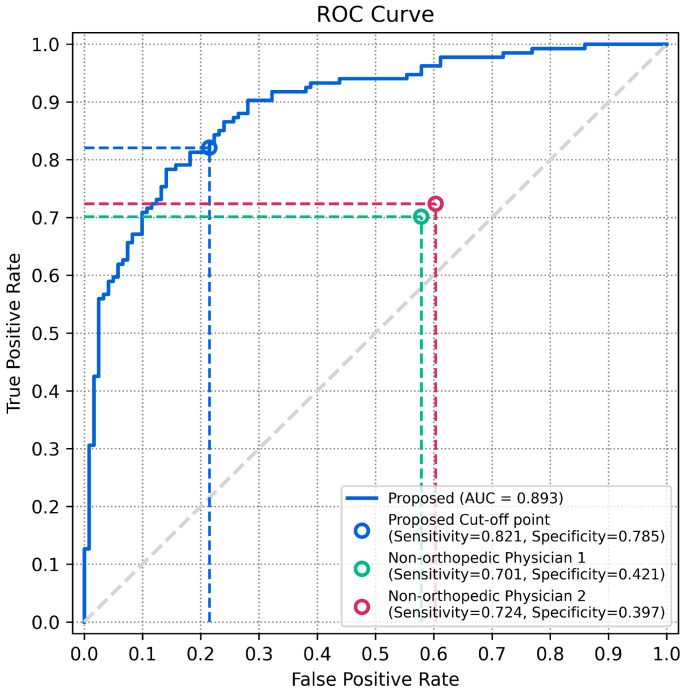
Comparison of physician evaluations on the ROC curve (non-orthopedic physician 1: physical medicine and rehabilitation; non-orthopedic physician 2: occupational and environmental medicine).

In our study, two physicians evaluated the presence or absence of knee effusion on the test-set radiographs. One had 7 years of experience in rehabilitation medicine and the other had 5 years in occupational and environmental medicine. The comparison of classification metrics and ROC curves between these physicians and our method is presented in Table 3 and Figure 5. Our method showed accuracy, sensitivity, and specificity scores of 0.803 (95% CI, 0.749–0.850), 0.820 (95% CI, 0.753–0.880), and 0.785 (95% CI, 0.710–0.851), respectively. In contrast, Physician 1 showed lower performance, with scores of 0.568 (95% CI, 0.505–0.630), 0.701 (95% CI, 0.626–0.776), and 0.421 (95% CI, 0.330–0.512). Physician 2 had scores of 0.568 (95% CI, 0.505–0.630), 0.723 (95% CI, 0.641–0.798), and 0.396 (95% CI, 0.314–0.479). All metrics showed significantly better performance for the proposed method compared to the two non-orthopedic physicians (*p* < 0.05).

### 3.3. Qualitative Results of the Classification Models

**Figure 6:**
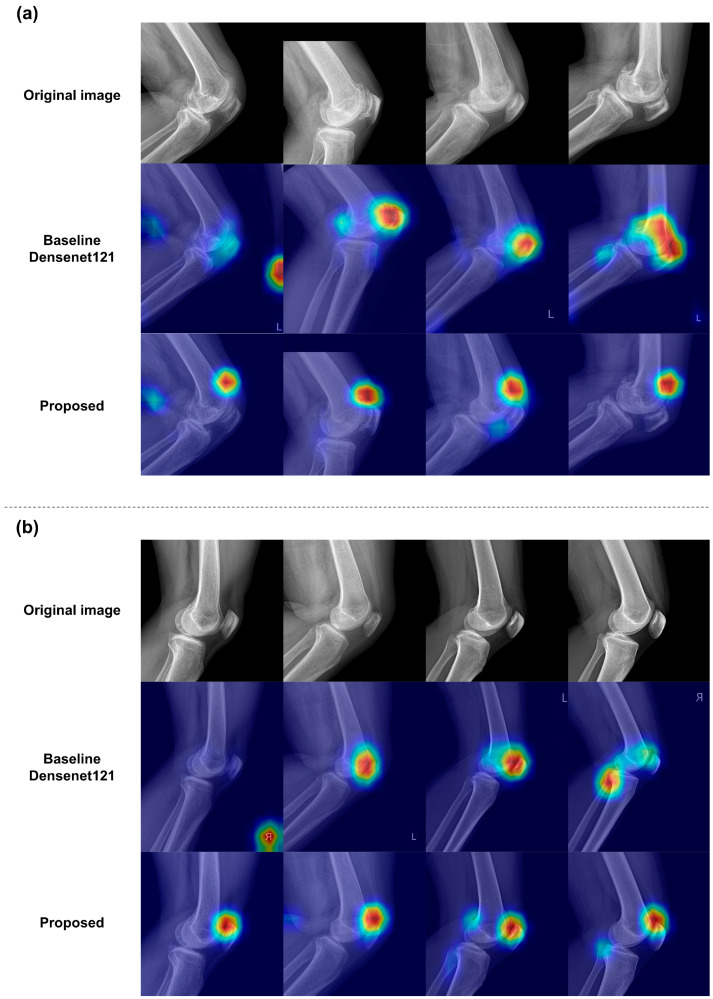
Visualization results using Eigen-CAM: (**a**) true positive cases; (**b**) true negative cases. The highly important features considered by the model for prediction are highlighted in red.

By applying a trained classification model that uses a binary label to indicate the presence of effusion, we generated Eigen-CAM images that highlight the effusion areas. These Eigen-CAM images emphasize regions related to effusion, typically located in the upper region of the knee joint. Figure 6 demonstrates the qualitative results, comparing Eigen-CAM with and without the knee structure-aware preprocessing, showing where the model identifies and emphasizes key areas using a heatmap. Additional results are illustrated in Supplement S4, and the results of comparing different CAM methodologies are shown in Supplement S5.

## 4. Discussion

In this study, we proposed a novel method for classifying the absence or presence of knee effusion in radiographs. By applying the proposed method, the model’s performance significantly improved, with an AUC of 0.892 compared to 0.821 for the model without our method. Additionally, our method significantly outperformed two non-orthopedic physicians in terms of accuracy, sensitivity, and specificity, achieving scores of 0.803, 0.820, and 0.785, respectively. These results demonstrate the potential of AI to facilitate the early and accurate classification of knee effusions.

Our findings reveal that while DenseNet121 has already shown robust performance in various clinical studies [37,38], our novel approach enhances the model’s ability to discern the presence or absence of knee effusion in radiographs. The proposed preprocessing method optimizes the input data to enhance image features important for identifying disease-specific conditions, focusing on knee regions related to effusion, such as the patella. In X-ray images, effusion can be very subtle and difficult to detect compared to MRI scans [10], even in patients with the disease. Therefore, it was necessary to utilize anatomically clear body structures for more robust standardization of the images and FoV. Accordingly, we devised a preprocessing method that detects the patella in lateral knee X-rays to precisely locate regions where effusion is likely to occur. This enables the model to perform more precise and accurate feature extraction and classification. Both qualitative and quantitative analysis showed that the preprocessing allowed for more nuanced interpretations of subtle clinical signs of effusion.

Currently, X-ray imaging is a major tool for the initial diagnosis of diseases due to its relatively low cost, minimal radiation exposure, and faster acquisition time [39]. Therefore, diagnosis of knee joint disorders is widely based on X-ray images [12]. One critical condition of knee joint disorders is effusion, which occurs outside the bones of the knee and can indicate other abnormalities within the joint [40]. However, visually identifying effusion in X-ray images is challenging, especially in the early stages, making it particularly difficult for non-orthopedic physicians [41]. In our physician evaluation, the results show that our model achieved higher diagnostic accuracy compared to non-orthopedic physicians. This may be due to two main reasons: First, the physicians involved in the study lacked specialized knowledge and experience in X-ray image interpretation, as they were not orthopedic surgeons familiar with arthroscopic surgery. Second, the dataset used in this study primarily consisted mostly of early-stage effusions, which can be more subtle and ambiguous to diagnose. Nevertheless, the AI model provided more accurate diagnoses because it has a superior ability to selectively focus on, interpret, and classify the unique patterns presented by effusions. Therefore, AI models can be used as a supportive computer-aided diagnosis system in other departments where diagnosing knee effusion is challenging for non-orthopedic surgeons.

Moreover, we were able to visualize the areas on which the AI model concentrated during effusion prediction by employing Eigen-CAM. The areas highlighted by Eigen-CAM accurately indicate the regions where effusion is present. This indicates that the model recognizes the visual patterns associated with the features of effusion locations. Nonetheless, effusion can be challenging to accurately capture using Eigen-CAM due to its blurred appearance compared to surrounding tissues and the unclear structure of the quadriceps tendon. However, Eigen-CAM emphasized the posterior quadriceps tendon and anterior patella, indicating that the model could consider thickened or indented areas of the femur or the synovial membrane as significant indicators. It might also consider the condition of the suprapatellar fat pad compressed by effusion fluid as a key factor in predicting the presence or absence of effusion. This visual interpretation offers insights into the model’s decision-making process based on specific anatomical structures and features, helping clinicians trust and effectively use AI predictions. Additionally, determining the presence of effusion heavily depends on the clinician’s experience. Therefore, Eigen-CAM can play an educational role by using visualization to help less experienced clinicians better understand the clinical signs of effusion.

The proposed methodology demonstrates promising clinical applicability in detecting knee effusion. This condition is closely associated with musculoskeletal pathologies, making the diagnosis of effusion crucial. The model can be effectively utilized to diagnose conditions related to knee joint effusion, such as OA and anterior cruciate ligament (ACL) tears. Furthermore, the proposed preprocessing methodology could be applied to other knee pathologies, including meniscal tears, tibial plateau fractures, ligament injuries, and patellar disorders. Moreover, the model’s ability to focus on specific anatomical regions suggests its potential for diagnosing effusions in other joints, such as the talus in the ankle and the epicondyle in the elbow. This indicates that the model could be expanded into a useful tool for diagnosing a variety of joint-related diseases.

Additionally, the inference time of the proposed method is a vital component of this study. The proposed method performed predictions on 255 images in just 4.332 s (0.016 s per image), indicating its capability to provide rapid and accurate diagnoses. This rapid inference time significantly improves the clinical applicability of the model, especially in medical environments where timely diagnosis is essential for patient care and treatment. The AI model can provide highly accurate diagnoses in just a few seconds, greatly supporting medical professionals and streamlining the diagnostic process. This rapid assessment enables AI to screen patients who specifically need immediate attention from a physician.

Our study has several limitations: First, the comparison experiment between non-orthopedic physicians and AI involved a limited number of participants, making it difficult to generalize if our results are representative of all non-orthopedic physicians. Additionally, we did not conduct comparison experiments with orthopedic physicians who are experts in diagnosing effusion. Furthermore, a reader study will be necessary to assess the clinical utility of the developed computer-aided diagnosis system [42,43]. Second, despite utilizing data from multiple centers, we aggregated all the data and randomly partitioned it into training and test sets. Therefore, we did not perform external validation. To evaluate the generalization performance of our model, we plan to establish an external validation dataset. Our model must perform well across diverse clinical settings, including handling knee images with features such as surgical scars or the poor-quality images that were excluded from this study. Therefore, we aim to enhance the model’s effectiveness by testing its performance across various clinical conditions and anatomical regions. Third, while the Eigen-CAM provides a rough indication of the location of effusions, it does not reveal the specific interpretable features considered by the model in making a diagnosis. Therefore, our future work aims to develop a model that uses a large language model (LLM) guide to explain, in text, the reasons for diagnosing effusion or normal conditions [44,45].

## 5. Conclusions

This study demonstrated the capabilities of the proposed deep learning model in diagnosing knee effusion, with significantly better performance than both the state-of-the-art deep-learning-based model and non-orthopedic physicians. The developed computer-aided diagnosis system based on the proposed method would greatly help in accurately and rapidly screening patients with effusion, aided by the interpretable visualization map.

## Figures and Tables

**Table 1:** The demographic information and acquisition parameters of multi-center images.

Characteristics	Hospital A (S.T.) (*n* = 280)	Hospital B (S.S.T.) (*n* = 233)	Hospital C (G.S.T.) (*n* = 450)	Hospital D (C.T. Hospital) (*n* = 450)
**Sex**				
Male	118	67	189	185
Female	162	166	261	265
**Age (mean ± SD)**	62 ± 4	62 ± 5	62 ± 2	63 ± 3
**Number of Image (disease statue)**				
Normal	168 (60%)	139 (59.7%)	150 (33.3%)	150 (33.3%)
Abnormal	112 (40%)	94 (40.3%)	300 (66.7%)	300 (66.7%)
**X-ray parameter**				
Tube potential (kVp)	60	60–70	60	60
Tube intensity (mA)	100	100	100	100
Exposure time (s)	0.125	0.300	0.125	0.125
**Focus to detector distance (cm)**				
Supine	85	100	100	100
Erect	78	100	100	100

S.T., Sungmo Top Orthopedics; Hospital A, S.S.T., Songpa Samsung Top Orthopedics; Hospital B, G.S.T., Guro Samaung Top Orthopedics; Hospital C, C.T. Hospital; Chungdam Top Orthopedics; Hospital D.

**Table 2:** Comparison of each method’s performance for classification; the highest values are bold-faced.

Metric (±95% CI)	DenseNet121	Proposed Method	*p*-Value ^†^
AUC	0.821 (0.770–0.872)	**0.892** (0.853–0.931)	<0.001
Accuracy	0.764 (0.707–0.815)	**0.803** (0.749–0.850)	0.133
Sensitivity	0.753 (0.686–0.835)	**0.820** (0.753–0.880)	0.052
Specificity	0.776 (0.619–0.785)	**0.785** (0.710–0.851)	1.000

^†^ *p*-values were calculated by DeLong’s test in AUC and McNemar’s test for the other metrics. CI, confidence interval.

**Table 3:** Comparison chart between the proposed method and physician evaluations. The highest values are bold faced (non-orthopedic physician 1: physical medicine and rehabilitation, non-orthopedic physician 2: occupational and environmental medicine).

Metric (±95% CI)	Proposed Method	Non-Orthopedic			
		Physician 1	*p*-Value ^†^	Physician 2	*p*-Value ^†^
Accuracy	**0.803** (0.749–0.850)	0.568 (0.505–0.630)	<0.001	0.568 (0.505–0.630)	<0.001
Sensitivity	**0.820** (0.753–0.880)	0.701 (0.626–0.776)	0.020	0.723 (0.641 –0.798)	0.048
Specificity	**0.785** (0.710–0.851)	0.421 (0.330–0.512)	<0.001	0.396 (0.314–0.479)	<0.001

^†^ *p*-values were calculated by McNemar’s test. CI, confidence interval.

## Data Availability

The data presented in this study are available on request from the corresponding author. The data are not publicly available due to patient privacy.

## Supplementary Materials

The following supporting information can be downloaded at: https://www.mdpi.com/article/10.3390/diagnostics14171900/s1, Figure S1: Comparison of the Visibility of Effusion in MRI and X-ray Images of the Knee from the Same Patient: (a) knee effusion captured by radiograph, (b) the same effusion captured by MRI, Figure S2: Comparison of image cropping techniques and the proposed method: The case 2 and case 4 are images where the effusion area and the knee shape have been cropped out, Figure S3: Results of classical segmentation algorithms, Figure S4: Procedure of knee structure-aware image preprocessing: (a) original image, (b) region growing, (c) translation, (d) padding: resize and centering for patella size matching, (e) cropping, Figure S5. Results for each scenario (the red box represents the original image, and the blue box represents the translated image), Figure S6: Visualization results using Eigen-CAM: (a) false positive cases, (b) false negative cases, Figure S7: Comparison results of visualizations among different CAM methods; Table S1. Comparison of the performance of image center point based cropping methods and the proposed method for classification., Table S2. Response times for each module in our proposed method, Table S3. Performance of baseline model. The highest values are bold faced., Table S4. Results with five fold cross-validation. References [27,29,30,31,32,33,34,40,46,47] are cited in the Supplementary Materials.

## References

[1] Hill C.L., Gale D.C., Chaisson C.E., Skinner K., Kazis L., Gale M.E., Felson D.T. (2001). Knee effusions, popliteal cysts, and synovial thickening: Association with knee pain in osteoarthritis. J. Rheumatol..

[2] Calmbach W.L., Hutchens M. (2003). Evaluation of patients presenting with knee pain: Part I. History, physical examination, radiographs, and laboratory tests. Am. Fam. Physician.

[3] Cole B.J., Harner C.D. (1999). Degenerative arthritis of the knee in active patients: Evaluation and management. JAAOS J. Am. Acad. Orthop. Surg..

[4] Stratford P. (1982). Electromyography of the quadriceps femoris muscles in subjects with normal knees and acutely effused knees. Phys. Ther..

[5] Scanzello C.R., Goldring S.R. (2012). The role of synovitis in osteoarthritis pathogenesis. Bone.

[6] Chiba D., Ota S., Sasaki E., Tsuda E., Nakaji S., Ishibashi Y. (2020). Knee effusion evaluated by ultrasonography warns knee osteoarthritis patients to develop their muscle atrophy: A three-year cohort study. Sci. Rep..

[7] Bachman A.L. (1946). Roentgen diagnosis of knee-joint effusion. Radiology.

[8] Maricar N., Callaghan M.J., Parkes M.J., Felson D.T. (2016). Clinical assessment of effusion in knee osteoarthritis—A systematic review. Semin. Arthritis Rheum..

[9] Engelstad B.L., Friedman E.M., Murphy W.A. (1981). Diagnosis of joint effusion on lateral and axial projections of the knee. Investig. Radiol..

[10] Cecava N.D., Dieckman S., Banks K.P., Mansfield L.T. (2018). Traumatic knee injury: Correlation of radiographic effusion size with the presence of internal derangement on magnetic resonance imaging. Emerg. Radiol..

[11] Ehlke M. (2021). 3d Reconstruction of Anatomical Structures from 2d X-ray Images. Ph.D. Thesis.

[12] Tiulpin A., Thevenot J., Rahtu E., Lehenkari P., Saarakkala S. (2018). Automatic knee osteoarthritis diagnosis from plain radiographs: A deep learning-based approach. Sci. Rep..

[13] Kawathekar P.P., Karande K.J. Severity analysis of Osteoarthritis of knee joint from X-ray images: A Literature review. Proceedings of the 2014 International Conference on Signal Propagation and Computer Technology (ICSPCT 2014).

[14] Majidi H., Niksolat F., Anbari K. (2019). Comparing the accuracy of radiography and sonography in detection of knee osteoarthritis: A diagnostic study. Open Access Maced. J. Med. Sci..

[15] Saleem M., Farid M.S., Saleem S., Khan M.H. (2020). X-ray image analysis for automated knee osteoarthritis detection. Signal Image Video Process..

[16] Rana M., Bhushan M. (2023). Machine learning and deep learning approach for medical image analysis: Diagnosis to detection. Multimed. Tools Appl..

[17] Choi E., Park D., Son G., Bak S., Eo T., Youn D., Hwang D. (2023). Weakly supervised deep learning for diagnosis of multiple vertebral compression fractures in CT. Eur. Radiol..

[18] Shin H., Kim H., Kim S., Jun Y., Eo T., Hwang D. SDC-UDA: Volumetric unsupervised domain adaptation framework for slice-direction continuous cross-modality medical image segmentation. Proceedings of the IEEE/CVF Conference on Computer Vision and Pattern Recognition.

[19] Park D., Jang R., Chung M.J., An H.J., Bak S., Choi E., Hwang D. (2023). Development and validation of a hybrid deep learning–machine learning approach for severity assessment of COVID-19 and other pneumonias. Sci. Rep..

[20] Shin H., Park J.E., Jun Y., Eo T., Lee J., Kim J.E., Lee D.H., Moon H.H., Park S.I., Kim S. (2023). Deep learning referral suggestion and tumour discrimination using explainable artificial intelligence applied to multiparametric MRI. Eur. Radiol..

[21] Barshooi A.H., Amirkhani A. (2022). A novel data augmentation based on Gabor filter and convolutional deep learning for improving the classification of COVID-19 chest X-ray images. Biomed. Signal Process. Control.

[22] Nasser Y., El Hassouni M., Hans D., Jennane R. (2023). A discriminative shape-texture convolutional neural network for early diagnosis of knee osteoarthritis from X-ray images. Phys. Eng. Sci. Med..

[23] Rutherford D.J., Baker M. (2018). Knee moment outcomes using inverse dynamics and the cross product function in moderate knee osteoarthritis gait: A comparison study. J. Biomech..

[24] Gaj S., Yang M., Nakamura K., Li X. (2020). Automated cartilage and meniscus segmentation of knee MRI with conditional generative adversarial networks. Magn. Reson. Med..

[25] Astuto B., Flament I., Namiri N.K., Shah R., Bharadwaj U., MLink T., DBucknor M., Pedoia V., Majumdar S. (2021). Automatic deep learning–assisted detection and grading of abnormalities in knee MRI studies. Radiol. Artif. Intell..

[26] Huhtanen J.T., Nyman M., Doncenco D., Hamedian M., Kawalya D., Salminen L., Sequeiros R.B., Koskinen S.K., Pudas T.K., Kajander S. (2022). Deep learning accurately classifies elbow joint effusion in adult and pediatric radiographs. Sci. Rep..

[27] Redmon J., Divvala S., Girshick R., Farhadi A. You only look once: Unified, real-time object detection. Proceedings of the IEEE Conference on Computer Vision and Pattern Recognition.

[28] Deng J., Dong W., Socher R., Li L.J., Li K., Fei-Fei L. Imagenet: A large-scale hierarchical image database. Proceedings of the 2009 IEEE Conference on Computer Vision and Pattern Recognition.

[29] Simonyan K., Zisserman A. (2014). Very deep convolutional networks for large-scale image recognition. arXiv.

[30] He K., Zhang X., Ren S., Sun J. Deep residual learning for image recognition. Proceedings of the IEEE Conference on Computer Vision and Pattern Recognition.

[31] Huang G., Liu Z., Van Der Maaten L., Weinberger K.Q. Densely connected convolutional networks. Proceedings of the IEEE Conference on Computer Vision and Pattern Recognition.

[32] Tan M., Le Q. Efficientnet: Rethinking model scaling for convolutional neural networks. Proceedings of the 36th International Conference on Machine Learning, ICML.

[33] Dosovitskiy A., Beyer L., Kolesnikov A., Weissenborn D., Zhai X., Unterthiner T., Dehghani M., Minderer M., Heigold G., Gelly S. (2020). An image is worth 16x16 words: Transformers for image recognition at scale. arXiv.

[34] Muhammad M.B., Yeasin M. Eigen-cam: Class activation map using principal components. Proceedings of the 2020 International Joint Conference on Neural Networks (IJCNN).

[35] DeLong E.R., DeLong D.M., Clarke-Pearson D.L. (1988). Comparing the areas under two or more correlated receiver operating characteristic curves: A nonparametric approach. Biometrics.

[36] McNemar Q. (1947). Note on the sampling error of the difference between correlated proportions or percentages. Psychometrika.

[37] Belaid O.N., Loudini M., Nakib A. Brain tumor classification using DenseNet and U-net convolutional neural networks. Proceedings of the 2024 8th International Conference on Image and Signal Processing and their Applications (ISPA).

[38] Pattanaik R.K., Mishra S., Siddique M., Gopikrishna T., Satapathy S. (2022). Breast Cancer Classification from Mammogram Images Using Extreme Learning Machine-Based DenseNet121 Model. J. Sens..

[39] Vabo S., Kjerstad E., Hunskaar S., Steen K., Brudvik C., Morken T. (2023). Acute management of fractures in primary care-a cost minimisation analysis. BMC Health Serv. Res..

[40] Johnson M.W. (2000). Acute knee effusions: A systematic approach to diagnosis. Am. Fam. Physician.

[41] Halbreiner U., Scariano V., Suppnig A., Haimburger E., Suppanz M. (2023). How do Trained and Prospective Physiotherapists and Radiologic Technologists Face Knee Joint Effusion Profession-Specifically and Interdisciplinary?—A Cross-Sectional Study. J. Orth. Clin. Res..

[42] Radiopaedia.org X-ray Interpretation: Knee Injuries. https://radiopaedia.org/courses/x-ray-interpretation-knee-injuries/pages/2042#1.

[43] Kiraly A.P., Cunningham C.A., Najafi R., Nabulsi Z., Yang J., Lau C., Ledsam J.R., Ye W., Ardila D., McKinney S.M. (2024). Assistive AI in Lung Cancer Screening: A Retrospective Multinational Study in the United States and Japan. Radiol. Artif. Intell..

[44] He P., Chen W., Bai M.Y., Li J., Wang Q.Q., Fan L.H., Zheng J., Liu C.T., Zhang X.R., Yuan X.R. (2023). Clinical Application of Computer-Aided Diagnosis System in Breast Ultrasound: A Prospective Multicenter Study. World J. Surg..

[45] Kaur D., Uslu S., Durresi M., Durresi A. (2024). LLM-Based Agents Utilized in a Trustworthy Artificial Conscience Model for Controlling AI in Medical Applications. Advanced Information Networking and Applications, Proceedings of the 38th International Conference on Advanced Information Networking and Applications (AINA-2024), Kitakyushu, Japan, 17–19 April 2024.

[46] Selvaraju R.R., Cogswell M., Das A., Vedantam R., Parikh D., Batra D. Grad-cam: Visual explanations from deep networks via gradient-based localization. Proceedings of the IEEE International Conference on Computer Vision.

[47] Jiang P.T., Zhang C.B., Hou Q., Cheng M.M., Wei Y. (2021). LayerCAM: Exploring Hierarchical Class Activation Maps for Localization. IEEE Trans. Image Process..

