# Supplementary material for: Deep Learning-Based Joint Effusion Classification in Adult Knee Radiographs: A Multi-Center Prospective Study
Source: Diagnostics (Basel). 2024 Aug 29;14(17):1900. doi: 10.3390/diagnostics14171900 (PMC11394442; doi:10.3390/diagnostics14171900)
Supplement: Supplementary file 1 [file diagnostics-14-01900-s001.zip › diagnostics-3139033-supplementary.pdf]

(Supplement) Deep Learning-Based Joint Effusion Classification in Adult Knee Radiographs: A Multi-Center Prospective Study

Supplement S1

Effusion in the knee joint is commonly associated with conditions such as osteoarthritis (OA), microfractures, cruciate ligament injuries, meniscus damage, infections, synovitis, and tumors [1]. This effusion can often be observed on X-ray examinations, which are the most commonly performed diagnostic tests and it serve as crucial biomarkers related to OA. A precise anatomical understanding of the knee, integrated with radiographic techniques, is essential for optimal patient management, allowing for accurate and timely diagnoses. However, effusions are more clearly visible on magnetic resonance imaging (MRI) scans and are less visible on X-ray images. An example of how effusion appears on an MRI scan and an X-ray image can be seen in Figure S1. Despite this, assessing effusion on X-ray images before proceeding to MRI scans remains important from a cost-effectiveness standpoint in current clinical practice.

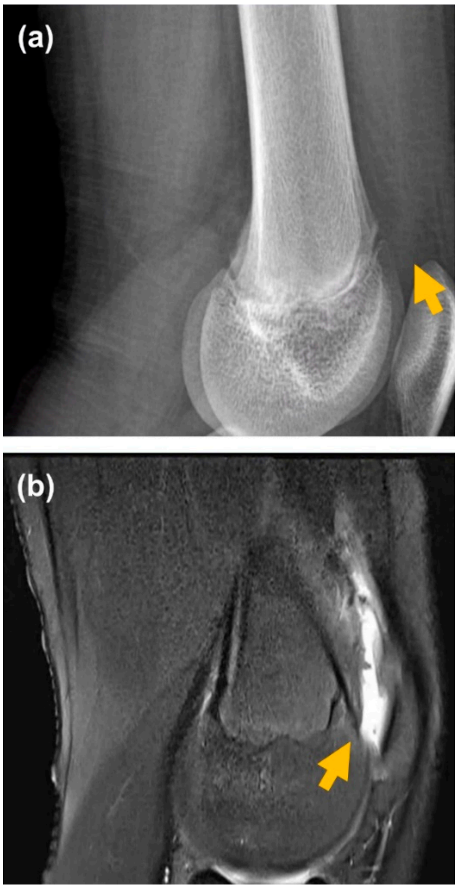

Figure S1. Comparison of the Visibility of Effusion in MRI and X-ray Images of the Knee from the Same Patient: (a) knee effusion captured by radiograph, (b) the same effusion captured by MRI

Supplement S2

S2.1. Experiment on image cropping methods and classical segmentation techniques for patella detection

We aimed to crop the regions containing knee effusion based on the anatomical structure of the patella. While it is possible to apply deep learning models such as YOLOv8 for this purpose, we conducted experiments where we cropped the images based on the center point of each image and experimented with classical segmentation methodologies including the level set and the fast-marching methods.

S2.1.1. Image center-based cropping

Due to the original images are smaller than the specified size of 1,600×1,600 in our methodology, we cropped the images to a size of 1,100×1,100 based on their center. After performing center-based

cropping and training the model, the performance decreased. The results of the center-based cropping are shown in Figure S2, and the test results with image cropping are presented in Table S1. This performance decline seems to be caused by cropping certain images in the dataset, especially ones containing important information, such as parts of the effusion or knee (e.g., parts of the knee shape of the patella region). Approximately 41.92% (537 cases out of 1,281 cases) of the total images were affected by this issue, preventing the model from learning the key anatomical structures needed to identify the shape of the knee or effusion.

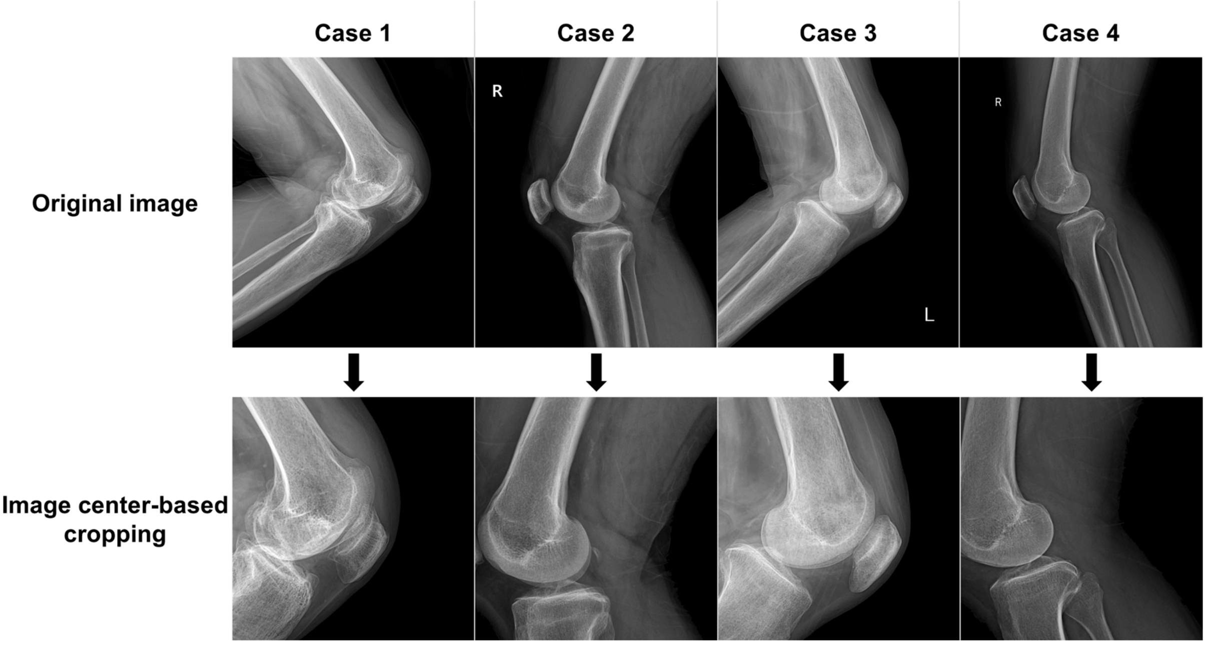

Figure S2. Comparison of image cropping techniques and the proposed method: The case 2 and case 4 are images where the effusion area and the knee shape have been cropped out.

Table S1. Comparison of the performance of image center point based cropping methods and the proposed method for classification.

Metric	Image center-based cropping	Proposed method
AUC	0.817	0.892
Accuracy	0.752	0.803
Sensitivity	0.783	0.820

Specificity	0.719	0.785

S2.1.2. Classical segmentation techniques

We performed classical segmentation algorithms, such as level set and fast-marching methods. These two classical segmentation methods produced inconsistent and coarse segmentation masks around the approximate patellar region, as illustrated in Figure S3. Subsequently, we applied the image size of 1,100×1,100 on the sets where masks were generated using both methods. Generally, cropping was performed within the range where effusion was included, as shown in Case 1 of Figure 3. However, there were instances, such as in Case 2 and Case 3, where the knee shape was not captured or the area where effusion was located was cut off. These issues occurred in 6.32% (80 cases out of 1,264 cases) for the level set method and 9.17% (116 cases out of 1,264 cases) for the fast-marching method. These algorithms rely on intensity-based curve connections, making it challenging to achieve clear object segmentation in X-ray images due to the similar intensity values in knee regions. Moreover, both methods require the specification of a seed point, which limits their ability to be fully automated. As a result, we determined that these algorithms are not suitable for cropping the knee regions, especially when effusion is present.

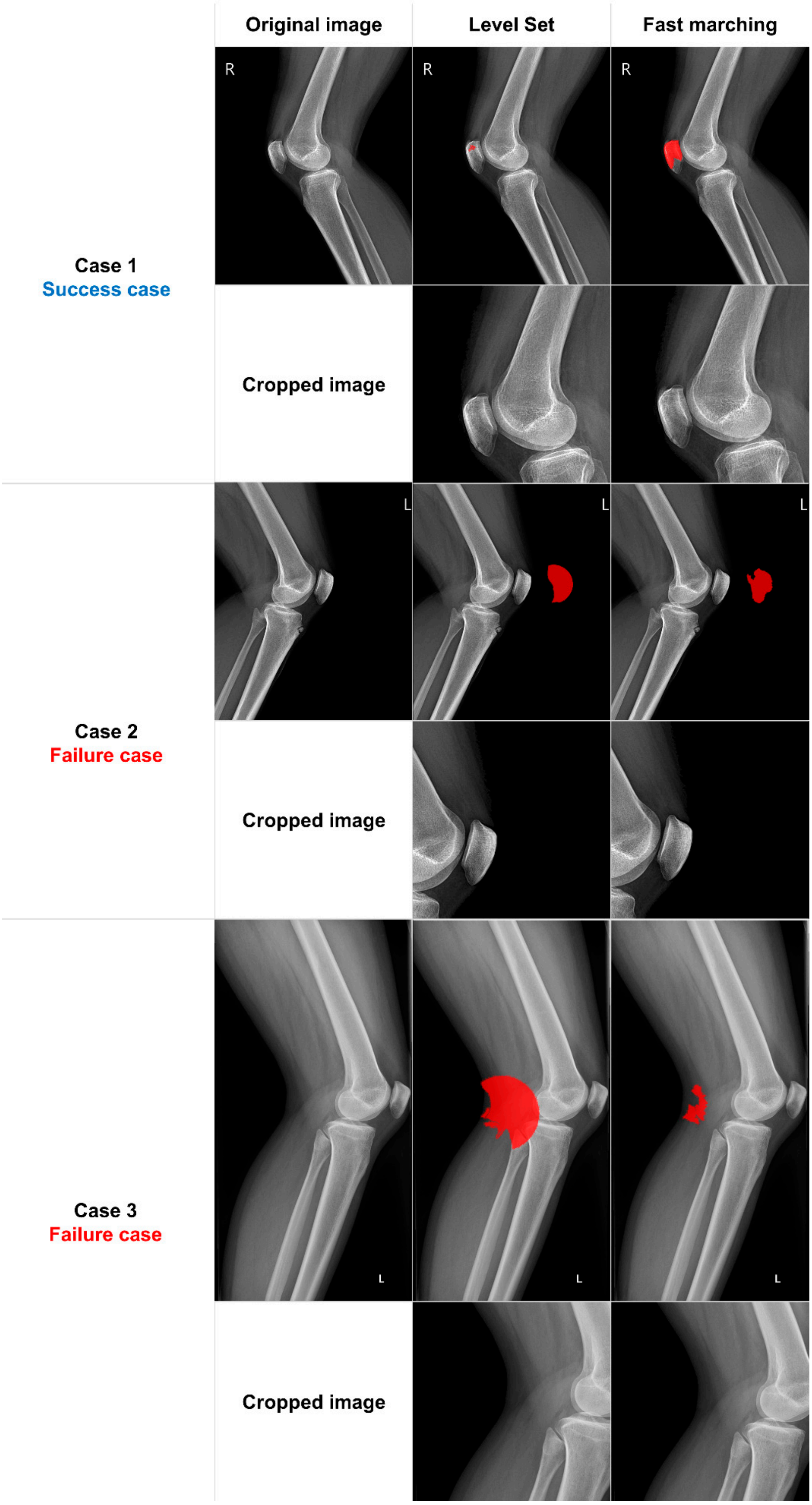

Figure S3. Results of classical segmentation algorithms

For these reasons, we decided to use YOLOv8 for the following reasons. YOLOv8 requires a training dataset with bounding box (bbox) labeling, but the annotation process only takes about 2 seconds per image. The training time for YOLOv8 takes about 15 minutes. The YOLO series models support real-time detection and are relatively lightweight compared to other detection models, making them resource efficient. In Table S2, we measured and compared the response times of each module used in our study, with YOLOv8 showing a response time of just 0.138 seconds, accounting for only 13.16% of the total time.

Therefore, using YOLOv8 for patella detection is an appropriate choice for pre-cropping, as it imposes minimal resource burden and provides more accurate detection compared to other algorithms.

Table S2. Response times for each module in our proposed method

Module	Time (s)
**Region growing**	0.290
**Detection (YoLov8)**	0.138
**Preprocessing**	0.093
**Classification (DenseNet121)**	0.015
**CAM (Eigen-CAM)**	0.511
**Total**	1.047

S2.2. Knee structure-aware image preprocessing

We developed a preprocessing strategy to address variation in field of view (FoV) and intensity levels in X-ray images collected from various institutions. Firstly, we employed a region-growing technique to isolate the knee by masking out all background elements outside the knee region, setting their values to zero. This effectively tackled the issue of intensity variation. Secondly, we constructed a detection model based on the patella of specific anatomical structure to crop the effusion area. Using the patella bbox labels from the training data, we trained a patella detection model using the YOLO [2] v8 architecture. To ensure that the knee was consistently oriented across all images, we assessed the sum of pixel values on the left and right sides of the bbox's central coordinates. If the left pixel sum was higher, the image was flipped, and we recorded whether a flip occurred. Subsequently, we calculated the average center of the bboxes to align the knees in the same position across all datasets. To standardize the average FoV, we scaled the data based on the smallest bbox and added zero padding to center the images after resizing. We then cropped all data to a uniform size of 1600×1600 pixels based on the average bbox center. This standardization ensured a consistent FoV and preserved effusion area information without distortion. Following this, if an image had been flipped during the knee alignment step, we used the recorded flip information to revert the knee to its original orientation. The results of our preprocessing method are presented in Figure S4. Moreover, to enhance robustness against rotational variance, we implemented image augmentation techniques, including random rotations between -15 and 15 degrees and horizontal flipping with a 0.5 probability, to increase data diversity.

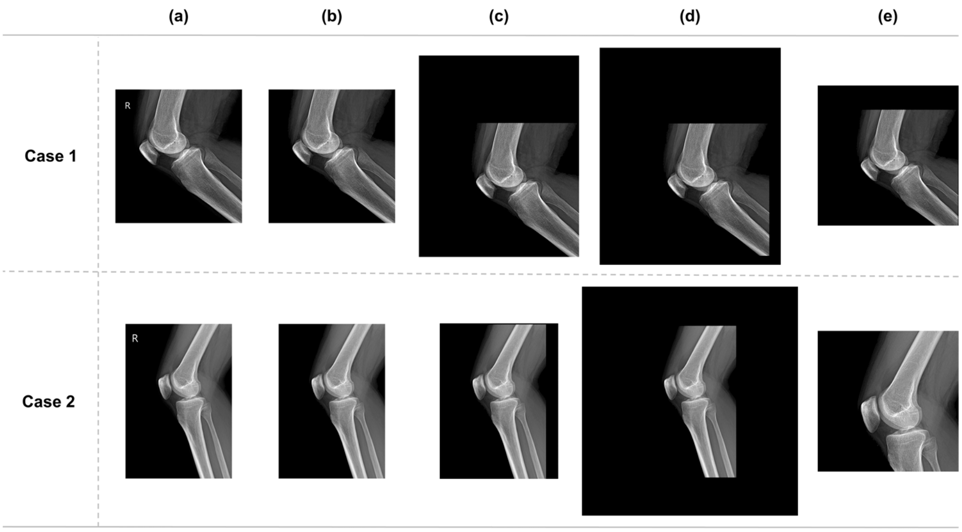

Figure S4. Procedure of knee structure-aware image preprocessing: (a) original image, (b) region growing, (c) translation, (d) padding: resize and centering for patella size matching, (e) cropping

S2.2.1 Detailed explanation of knee structure-aware image preprocessing

Our preprocessing procedure is primarily designed to accurately detect the patella in knee images and capture areas that include effusion. This approach aligns the field of view (FoV) for the region of interest (ROI) in multi-center data and considers the tissue structure around the patella, which plays a crucial role in detecting effusion. Therefore, our method aims to diagnose only the essential areas to improve the accuracy of effusion classification in lateral knee X-ray images. The standardized preprocessing is conducted according to the following four steps:

Step 1. Region growing and patella detection:

We detect the ROI for the patella using X-ray images. Initially, we apply a region growing technique, which identifies objects by expanding areas based on grayscale value similarities. This technique distinguishes object areas from the background. Subsequently, we use a pre-trained YOLOv8 model to detect the patella. In this process, we obtain an average target center of the bounding box (bbox) at (547, 1035), with a minimum bbox size of 120.19 in width and 205.88 in height.

Step 2. Center translation:

We translate the center of the patella detected by YOLOv8 in each image to the average target center determined in the previous step. Traditional padding methods often add padding of a fixed size to the edges of the image, which may not translate the patella's center as needed. To address this, we calculate a translation vector to align the patella's center coordinates with the specific average target center coordinate, and then apply padding based on the calculated translation. This step ensures that the patella's ROI is positioned at the average target center within the new image, thereby enhancing the consistency of subsequent analyses.

Step 3. Resizing and zero padding:

Given that multi-center data does not have a consistent FoV, we aimed to standardize the overall FoV and consistently adjust the ROI by aligning the patella bbox sizes. We calculate a dynamic scale factor for each image to adjust the patella bbox size to the minimum bbox without distorting the image, while maintaining the original aspect ratio. The resizing step involves calculating the scale factor based on the ratio of the reference box's width and height to determine the degree of adjustment. For example, we use the smaller ratio of the reference box's width or height as the scale factor. The transformed image size is adjusted using the scale factor, and uniform zero padding is applied to all sides of the image to standardize the patella bbox size. This approach allows the model to effectively handle images of various sizes.

Step 4. Image cropping:

Finally, we crop the resized image to 1600×1600 around the patella center to generate input images that are suitable for a neural network.

Each step of the preprocessing process is designed to provide standardized inputs for X-ray images of various sizes and views. The results of each step are shown in Figure S5.

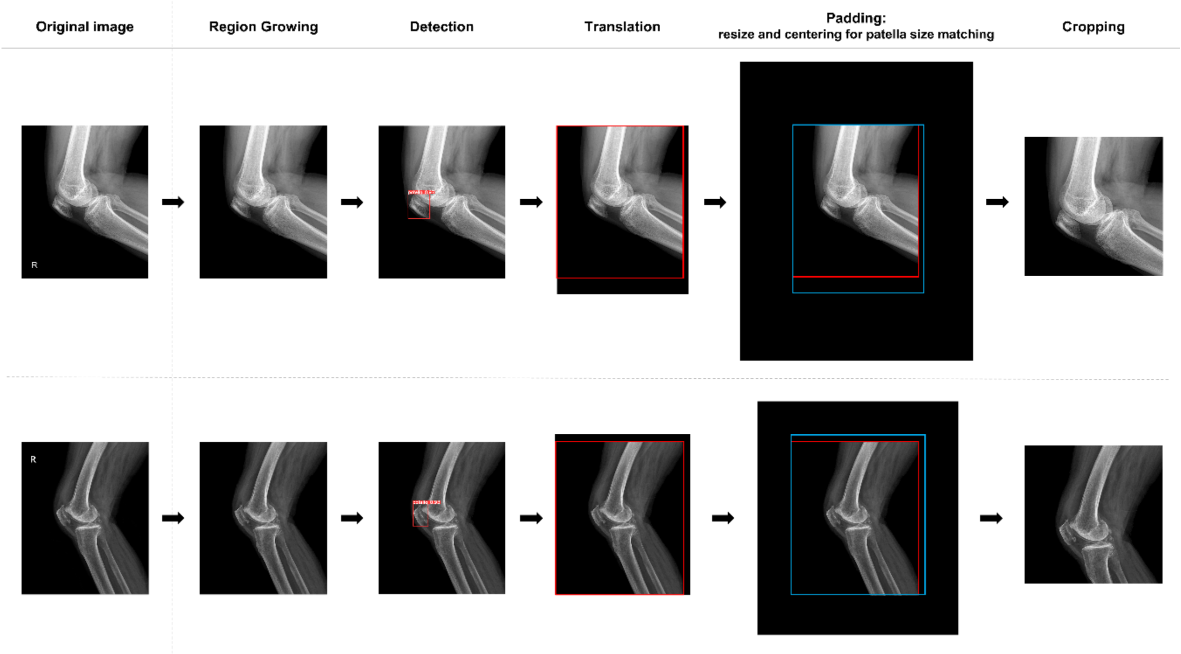

Figure S5. Results for each scenario (the red box represents the original image, and the blue box represents the translated image)

Supplement S3

To establish a baseline for model performance comparison, we trained the five models using original data. The performance comparison is presented in Table S3.

Table S3. Performance of baseline model. The highest values are bold faced.

Model	AUC score	Accuracy	Sensitivity	Specificity
VGG19 [3]	0.808	0.729	0.694	0.768
ResNet50 [4]	0.790	0.733	0.701	0.768
DenseNet121 [5]	**0.821**	**0.764**	**0.753**	0.776

EfficientNet B5 [6]	0.754	0.705	0.552	0.876
ViT [7]	0.809	0.749	0.671	**0.834**

Supplement S4

Figure S6 displays the false positive (FP) and false negative (FN) cases identified by the classification model when detecting effusion in the knee joint.

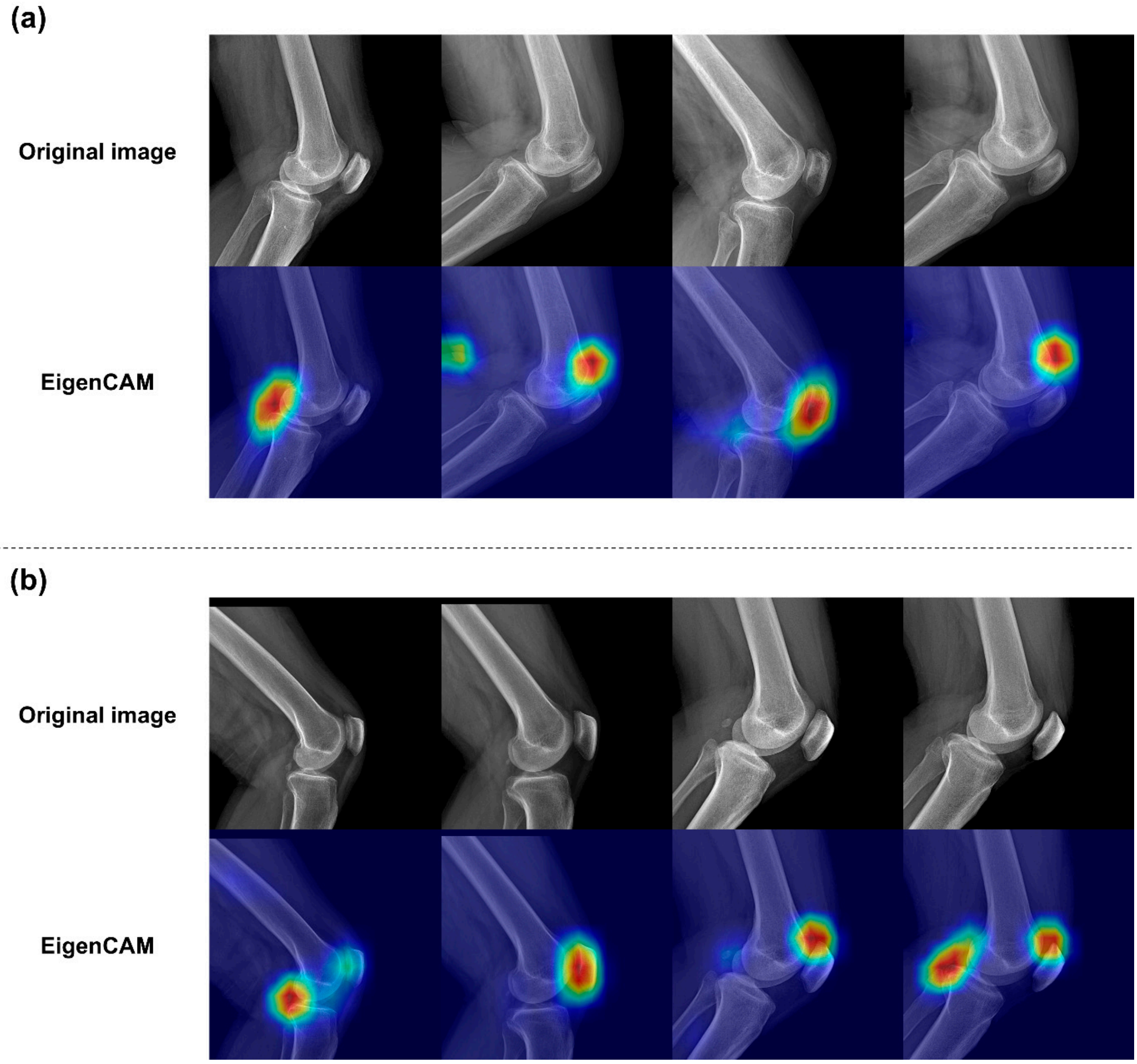

Figure S6. Visualization results using Eigen-CAM: (a) false positive cases, (b) false negative cases

Supplement S5

We aim to compare various class activation mapping (CAM) methods for true positive (TP) and true negative (TN) cases. This study compares the results of Eigen-CAM [8], Grad-CAM [9], and LayerCAM [10], which are presented in Figure S7.

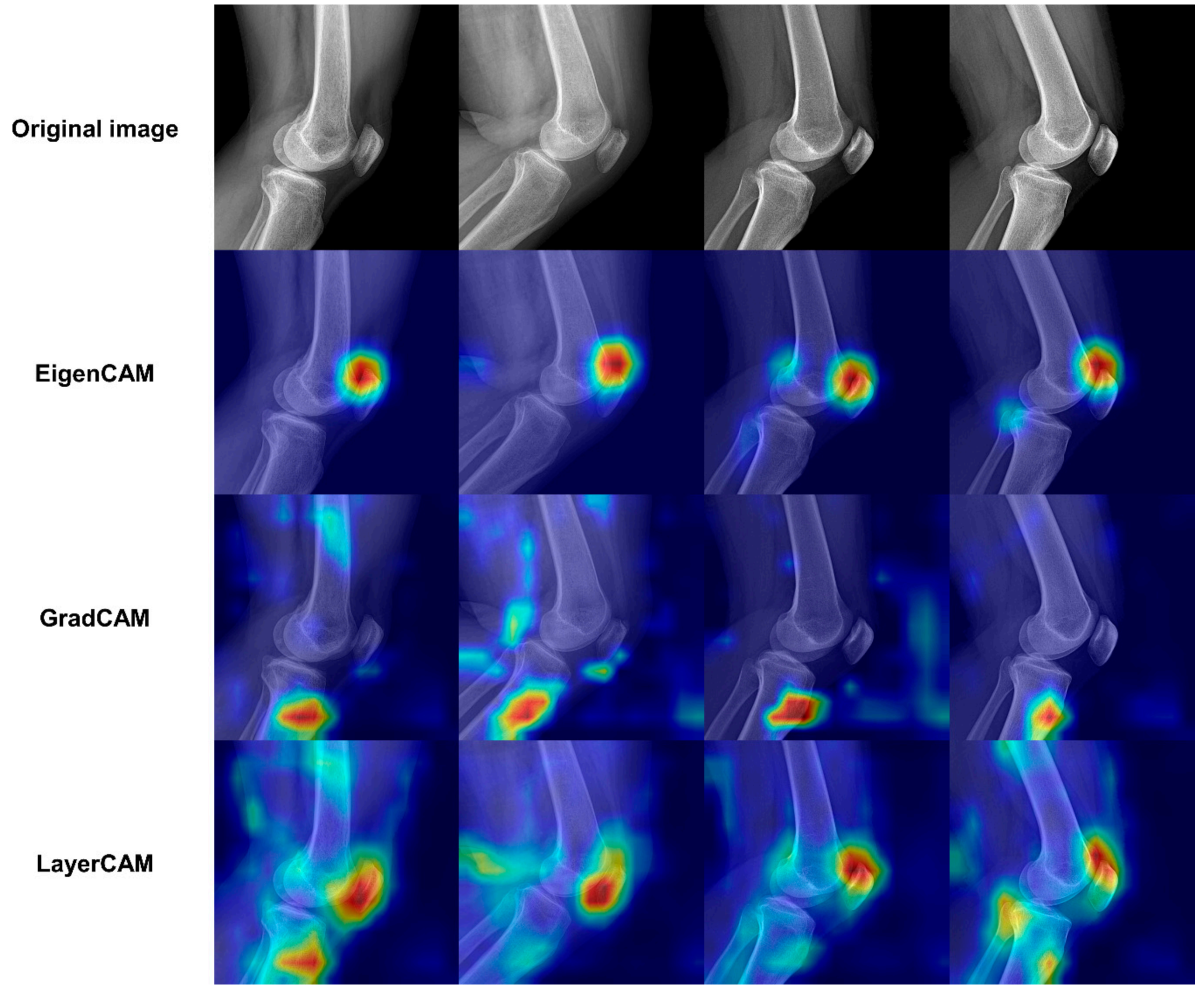

Figure S7. Comparison results of visualizations among different CAM methods

Supplement S6

The absence of external validation results is a clear limitation of this study. However, due to budget constraints, it is currently difficult to collect additional data. To ensure the reliability of our research results under these conditions, additional experiments were conducted using 5-fold cross-validation,

and the results are presented in Table S4.

Table S4. Results with five fold cross-validation

Folds	Metrics			
	AUC	Accuracy	Sensitivity	Specificity
1	0.885	0.760	0.940	0.561
2	0.873	0.800	0.731	0.876
3	0.871	0.796	0.723	0.876
4	0.874	0.776	0.858	0.685
5	0.886	0.784	0.925	0.628
**Average**	0.879	0.783	0.835	0.725

Reference

1. Johnson, M.W. Acute knee effusions: A systematic approach to diagnosis. *Am. Fam. Physician* **2000**, *61*, 2391–2400.
2. Redmon, J.; Divvala, S.; Girshick, R.; Farhadi, A. You only look once: Unified, real-time object detection. In Proceedings of the IEEE Conference on Computer Vision and Pattern Recognition, Las Vegas, NV, USA, 27–30 June 2016.
3. Simonyan, K.; Zisserman, A. Very deep convolutional networks for large-scale image recognition. *arXiv* **2014**, arXiv:1409.1556.
4. He, K.; Zhang, X.; Ren, S.; Sun, J. Deep residual learning for image recognition. In Proceedings of the IEEE Conference on Computer Vision and Pattern Recognition, Las Vegas, NV, USA, 27–30 June 2016.
5. Huang, G.; Liu, Z.; Van Der Maaten, L.; Weinberger, K.Q. Densely connected convolutional networks. In Proceedings of the IEEE Conference on Computer Vision and Pattern Recognition, Honolulu, HI, USA, 21–26 July 2017.
6. Tan, M.; Le, Q. Efficientnet: Rethinking model scaling for convolutional neural networks. In Proceedings of the 36th International Conference on Machine Learning, ICML, Long Beach, CA, USA, 9–15 June 2019.
7. Dosovitskiy, A.; Beyer, L.; Kolesnikov, A.; Weissenborn, D.; Zhai, X.; Unterthiner, T.; Dehghani, M.; Minderer, M.; Heigold, G.; Gelly, S.; et al. An image is worth 16x16 words: Transformers for image recognition at scale. *arXiv* **2020**, arXiv:2010.11929.
8. Muhammad, M.B.; Yeasin, M. Eigen-cam: Class activation map using principal components. In Proceedings of the 2020 International Joint Conference on Neural Networks (IJCNN), Glasgow, UK, 19–24 July 2020.
9. Selvaraju, R.R.; Cogswell, M.; Das, A.; Vedantam, R.; Parikh, D.; Batra, D. Grad-cam: Visual explanations from deep networks via gradient-based localization. In Proceedings of the IEEE International Conference on Computer Vision, Venice, Italy, 22-29 October, 2017.
10. Jiang, P.T.; Zhang, C.B.; Hou, Q.; Cheng, M.M.; Wei, Y. LayerCAM: Exploring Hierarchical Class Activation Maps for Localization. *IEEE Trans. Image Process.* **2021**, *30*, 5875–5888.
